# Educational difference in the prevalence of osteoporosis in postmenopausal women: a study in northern Iran

**DOI:** 10.1186/1471-2458-11-845

**Published:** 2011-11-03

**Authors:** M Maddah, SH Sharami, M Karandish

**Affiliations:** 1 Nutrition Research Center, Ahvaz Jundishapur University of Medical Sciences, P.O. Box: 41635-3197, Ahvaz, Rasht-Iran, Iran; 2 Department of Gynecology, School of Medicine, Guilan University of Medical Sciences, Rasht, Iran

**Keywords:** Educational levels, Osteoporosis, Postmenopausal women

## Abstract

**Background:**

Osteoporosis is the most common metabolic bone disease in the world and it is rapidly increasing in Iran. In this study the relationship between educational levels and osteoporosis was investigated among Iranian postmenopausal women.

**Method and subjects:**

Seven hundred and six women aged 50-75 years old were randomly recruited from urban (*n *= 440) and rural (*n *= 266) areas in Guilan. Osteoporosis was diagnosed by quantitative ultrasound technique and dual X-ray absorptiometry. Serum 25(OH) D3, body weight and height were measured in all subjects. Other data including age, educational level, menopause age, medications and history of illness were also collected.

**Results:**

We found that the prevalence of osteoporosis was significantly greater among women with low educational level than women with high educational status (18.0% vs 3.8% *P *< 0.0001). However, women with low educational level had higher mean serum level of vitamin D than women with high educational level. Osteoporosis was significantly more prevalent among women living in rural areas than women living in urban areas (19.1% v.s 13.3%, *P *< 0.0001).

**Conclusion:**

This study showed that educational level is associated with bone health in this population of postmenopausal women with significantly higher osteoporosis found in lower social groups. Therefore, we suggest that women with low social level should be carefully evaluated for signs of osteoporosis during routine physical examinations.

## Background

Osteoporosis is a major public health issue, and its incidence is expected to increase in association with the worldwide aging of the population [1-3].

For most diseases and overall mortality a social gradient exists [4,5] however the association of osteoporosis with social status has not been studied in Iran. Evidences show that peak bone mass among all age groups of Iranian population, is lower than European or American populations [6]. A high prevalence of fractures due to osteoporosis was reported, in some less developed areas in Iran especially in low income groups [7-9]. However, there is less information on relationship between educational levels and osteoporosis in Iranian population.

Guilan is a province in northern Iran which half of its population live in rural areas. The aim of this study was to investigate the relationship between educational levels and osteoporosis among a population of post-menopausal women in urban and rural areas in Guilan, northern Iran.

## Methods and subjects

The diagnosis of osteoporosis was carried out by quantitative ultrasound (QUS) technique using the Sahara Clinical Sonometer (Hologic Inc, Bedford, MA, USA) according to standardized protocol. The machine was daily calibrated with the physical phantom provided by the manufacturer. The outputs included broadband ultrasound attenuation (BUA), speed of sound (SOS) and a machine derived parameter: calcaneal bone mineral density (eBMD) in g/cm^2^, eBMD = 0.002592 × (BUA+SOS)-3.687. Then, subjects with positive results were confirmed by dual X-ray absorptiometry [10].

A blood sample was collected and transferred to a private laboratory (Razi Medical Laboratory, Rasht, Iran) for measuring of serum 25(OH) D. Serum 25(OH) D) was measured by radioimmunoassay using a commercial kit (BioSource Europe S.A. Rue del'Industrie, 8, B-1400 Nivelles, Belguim). In this study, the levels of serum 25(OH) D lower than 15 ng/ml was considered as vitamin D deficiency. Anthropometric measurements were performed in lightly dressed women without shoes in the morning. Body mass index (BMI) was calculated using the following equation: weight (kg)/[height (m)]^2^. In this study, based on schooling years, subjects were divided into low (< 12 years schooling) and high (≥ 12 years schooling) educational groups. In this study all subjects signed a consent form and the study protocol was approved by the ethical committee of Guilan University of Medical Sciences.

Statistical analysis: means, standard deviations and percentages were used to describe the data. Student *t *test and Chi 2 tests were used to compare the differences between two groups. All data were analyzed by Statistical Package for Social Science (SPSS 10.01 for windows, SPSS Inc^® ^headquarter, Chicago, IL, USA).

## Results

Table 1 shows the mean age, serum level of vitamin D and prevalence of osteoporosis by educational levels. The results indicated that BMI were not different between low and high educated groups. Furthermore, less educated women were older and more likely to be rural resident than higher educated women. Osteoporosis was significantly more prevalent in low educational group than in high educational group. Women with low educational level had higher mean serum level of vitamin D than women with high educational level. Urban and rural differences in the prevalence of osteoporosis and vitamin D status were presented in Table 2. Our findings showed that rural women had greater serum level of vitamin D than urban women and vitamin D deficiency (serum level less than 15 ng/ml) were more prevalent among urban women than rural women (49.4% and 27.9% *p *< 0.0001). Osteoporosis was significantly more prevalent among women living in rural areas than women living in urban areas (Table 2).

**Table 1:** Prevalence of osteoporosis, mean age, serum levels of vitamin D, urban/rural residence and BMI among women by their level of education

Variables	≥ 12 years schooling (*n *= 593)	< 12 years schooling (*n *= 113)	*P*-value
Age (years)	62.9 ± 6.9	56. 7 ± 5.9	0.0001

Osteoporosis (%)	18.0	3.8	0.0001

Vitamin D3 (ng/ml)	20.3 ± 13.6	15.9 ± 5.9	0.001

BM I (kg/m^2^)	29.0 ± 5.3	29.8 ± 5.7	0.1

Urban resident (%)	56.9	97.7	0.0001

Rural resident (%)	43.1	2.3	0.0001

**Table 2:** Prevalence of osteoporosis, mean age, serum levels of vitamin D, and BMI among women by urban/rural residence

Variables	Urban (*n *= 440)	Rural (*n *= 266)	*P*-value
Age (years)	61.6 ± 7.1	62.17 ± 7.2	0.3

Osteoporosis (%)	13.3	19.1	0.0001

Vitamin D3 (ng/ml)	18.2 ± 13.2	22.3 ± 2.8	0.0001

BM I (kg/m^2^)	29.8 ± 5.3	28.0 ± 5.5	0.1

Low educated (%)	72.2	98.9	0.0001

High educated (%)	22.8	1.2	0.0001

## Discussion

Education is one of the most commonly used measures of socioeconomic status (SES) in epidemiological studies [11]. We found that post-menopausal women with low education were more likely to have osteoporosis than high educated women. Although the mean age of low educated women was greater than high educated women, the prevalence of osteoporosis among low educated women was approximately five times more than high educated women. This finding is concur with the findings of western countries indicating that low educated women are more prone to low density bone and osteoporosis than high educated women [12,13].

Many risk factors are associated with osteoporosis, including low peak bone mass, hormonal factors, use of certain drugs, cigarette smoking, low physical activity, low intake of calcium and vitamin D, and low BMI. Some of these risk factors are expected to be more prevalent in people with low educational levels, especially in developing countries. In Iran, the last National Nutrition and Dietary Survey showed that many Iranians, especially in low SES do not meet the Estimated Average Requirement for calcium [14]. We previously showed that only a small proportion of Iranian elderly women, especially in low educational levels, used calcium/vitamin D supplement [15]. There are evidence to indicate that in less developed regions of Iran, significantly higher rates of fracture are seen in lower income groups than higher income groups [7,8]. Further research is needed to understand the mechanism of these associations and how they may contribute to increased risk of osteoporosis in subgroups of a population.

These data showed that high educated women had lower serum vitamin D than low educated women. In general, Iranian women, as Muslim, have limited sun exposure due to their wearing habits especially among urban women. In this study, most of low educated women were living in rural areas. Such urban-rural differences in vitamin D status may be explained by living condition and housing status of people in urban and rural areas. While elderly women in urban areas are more likely to stay indoors, rural women usually work as farmer even in old ages, therefore, and they have more sun exposure than urban women.

In conclusion, this study showed that social inequalities play an important role in bone health, with significantly higher osteoporosis among lower social groups. These data indicate that osteoporotic fracture risk may be higher in post-menopausal women with lower social status in Iran. Therefore, it is suggested that women with low social status should be carefully evaluated for signs of osteoporosis during routine physical examinations.

## Competing interests

The authors declare that they have no competing interests.

## Authors' contributions

MM designed the study analyzed the data and wrote the paper. HS helped collecting data. MK helped collecting data and preparing the draft of the paper.

## Pre-publication history

The pre-publication history for this paper can be accessed here:

http://www.biomedcentral.com/1471-2458/11/845/prepub
